# Spontaneous and induced hyperplasia and neoplasia in the mouse lung.

**DOI:** 10.1038/bjc.1966.14

**Published:** 1966-03

**Authors:** P. M. Peacock, P. R. Peacock

## Abstract

**Images:**


### SPONTANEOUS AND INDUCED HYPERPLASIA AND NEOPLASIA IN THE MOUSE LUNG

P. M. PEACOCK* AND P. R. PEACOCK

*From the Cancer Research Department, Royal Beatson Memorial Hospital, Glasgow*

Received for publication December 20, 1965

SINCE Livingood (1896) first described a spontaneous pulmonary tumour in a mouse, much has been written on the histogenesis of this common tumour, yet it is still difficult to decide whether an early lesion should be classified as hyperplastic or neoplastic.

Since the introduction of line-bred strains of mice by Strong (1936) and their general use by research workers, genetic differences in susceptibility have been extensively investigated by Heston (1940) and many others. It is accepted that the susceptibility to spontaneous development of pulmonary tumours is high in A Strain and low in C57 Black, but that there is no essential difference between the type or range of tumours seen in different strains (Stewart, 1953).

As to the sites of origin and distribution of these tumours, most authors describe them as subpleural and as originating in the alveolar epithelium. As far as we know, there has been no evidence as to localising factors which cause one alveolus rather than another to develop neoplastic growth.

After surveying the histological evidence in many hundreds of spontaneous and induced lung tumours in mice of several strains, we have adopted a system of classification which provides objective standards for differential diagnosis between hyperplasia and neoplasia. We classify all tumours according to their points of origin, as " sub-pleural " or " other sites ".

The strains of mice examined were BALB/c, $C3H_f$, $RIII_f$, C57 Black, LX, and our heterozygous closed colony. No essential differences were found between the types of lesion in these strains though the incidence varied, as is well known. We found a single example of deep-seated spontaneous papillary adenoma in a male C57 Black mouse, 817 days old.

We also compared the induced and spontaneous pulmonary tumours in BALB/c mice in two widely different age groups exposed to different types of chemical carcinogens.

### ANATOMICAL CLASSIFICATION

### *Tumours of subpleural origin*

Tumours arising in alveoli in contact with the pleura can grow more readily into the lung than into and through the pleura. Consequently, such tumours, when seen early in their development, appear in microscopic section as wedge-shaped lesions with a slightly bulging base covered by the pleura and with the apex pointing in towards the centre of the lung (Fig. 1). This type of tumour originates in peripheral alveoli, often immediately below one of the numerous

* Present address: Royal Maternity Hospital, Glasgow.

pleural lymphoid follicles which have a diameter of 1 or 2 millimeters and form part of the normal lymphatic anatomy of the pleura (Peacock, 1965*a*). When hyperplasia or neoplasia originates in such subpleural sites the associated lymphatics are often engorged with apparently normal lymphocytes (Fig. 2 and 12), whereas the rest of the lung shows no such abnormality. It seems unlikely therefore that this association can be fortuitous. Selbie and Thackray (1948) mention variations in lymphocytic infiltration in association with pulmonary tumours in mice, but without attaching particular significance to their presence or anatomical situation.

In mice which show deposits of soot in the pleura, these correspond with the presence of soot-laden macrophages in the walls of the pleural lymphoid follicle and it has been our experience that occasionally a subpleural tumour appears as a white nodule surrounded by a black halo, due to compression of the associated follicle by the expanding tumour (Fig. 3 and 4).

*Tumours arising at other sites in the lung*

In contrast, tumours originating in the depth of the lung and growing symmetrically will very soon appear to be subpleural. With an organ of the size of the mouse lung, there is no point in any one of its five lobes more than about 3 mm. from the nearest pleural surface (Fig. 5*a*). It follows that a tumour need only reach a diameter of 2 to 3 mm. to appear to be subpleurally situated because of the ease with which the lung can be compressed between the tumour and the tough overlying pleura (Fig. 6).

With this in mind, we have compared two groups of BALB/c mice differing widely in age and in the type of carcinogenic exposure but showing the same range of hyperplastic and neoplastic lesions ; and histological material from several other experiments.

### MATERIALS AND METHODS

*Group* 1.—It has been shown that single injections of small quantities of carcinogens in newborn mice induce tumours at various sites relatively quickly (Pietra, Spencer and Shubik, 1959 ; Roe, Rowson and Salaman, 1961). In a modification of this technique, 111 mice, 6 to 24 hours old, were injected either subcutaneously or intraperitoneally once only with 0·5 ml. of a suspension of a carcinogenic polycyclic hydrocarbon in 0·1 per cent aqueous solution of sodium alginate. The concentration was calculated on an equimolecular basis so that each injection contained approximately 50 $\mu$g. hydrocarbon. The distribution of lung lesions is shown in Table I.

Seventy-six newborn mice were similarly injected with a suspension of polycyclic hydrocarbons considered to be non-carcinogens ; and 26 untreated mice were kept for control study. All were killed when 12 weeks old.

*Group* 2.—BALB/c mice of both sexes aged from 9 to 14 weeks were given daily in their drinking water isoniazid (INH) at the dosage level 1–2 mg. daily per 20 g. mouse. They died or were killed with chloroform at various time intervals thereafter until 2 years of age, when all the survivors were killed. The lungs of 26 mice so treated were compared with the lungs of 45 untreated controls matched for age. Animals which were sick for more than 24 hours were killed at whatever their age.

TABLE I.—*Incidence of Alveolar Hyperplasia in BALB/c Mice Surviving* 12 *Weeks after a Single Injection at Birth of Carcinogen in Alginate*

| | Subcutaneous injection | Intraperitoneal injection |
|---|---|---|
| 3,4-benzopyrene | 1/9 | 0/7 |
| 1,2 : 3,4-dibenzopyrene | 0/4 | 0/9 |
| 1,2 : 4,5-dibenzopyrene | 0/4 | 1/3 |
| 3,4 : 8,9-dibenzopyrene | 0/8 | 0/9 |
| 3,4 : 9,10-dibenzopyrene | 1/7 | 1/8 |
| 1,2-benzanthracene | 0/11 | 0/4 |
| 1,2 : 5,6-dibenzanthracene | 1/5 | 4/6 |
| 1,2 : 7,8-dibenzanthracene | 0/9 | *1/8 |

* Shows subpleural papillary adenoma formation also in one lesion, Fig. 12.
All foci of alveolar hyperplasia were separated from the pleura by normal lung tissue.
Results expressed as number of mice with lesions/number of mice injected.

*Group* 3.—This includes $C3H_f$ mice exposed to isoniazid and BALB/c mice exposed to 4-nitroquinoline-N-oxide (NQNO).

### *Post Mortem Examination*

Animals that were killed were immediately autopsied and those that were found dead were examined as soon as possible. The abdominal viscera were first examined and removed and the lung was then inspected through the diaphragm using a large mounted lens. Any lesions of the bases of the lungs could be seen through the diaphragm and their position noted. The pectoral muscles were then dissected off the thoracic wall and tumours situated in the anterior and lateral aspects of both lungs could be seen and their position noted in terms of the ribs and intercostal spaces. The diaphragm was punctured to collapse the lungs and 1 per cent formalin solution was injected through the trachea until the lungs assumed the position of full inflation in contact with the diaphragm. The trachea was ligated and the whole thorax removed for histological study. In some cases the whole thorax was decalcified and cut in serial section, but as a rule the lungs were removed from the thoracic cage, examined lobe by lobe for possible deep-seated tumours, and then transferred to the lid of a plastic petri dish ; the heart was removed and the inverted base of the petri dish was then pressed down gently on to the lungs to compress them to a thickness of about 2 mm. The lungs could thus be inspected from both sides against a bright transmitted light, when tumours appeared as translucent spots against the bright red of the normal lung tissue. In this way it is possible to recognise tumours of 1 mm. or more diameter in any part of the lung (Fig. 7*a* and *b*).

### *Histological Classification*

*Histology.*—Serial sections were prepared from all lungs and were studied by staining alternate ribbons, usually of four sections, with haematoxylin and eosin. Van Giesen's plus elastin stain, and sometimes Mallory or reticulum stains.

The earliest lesions noted were hyperplastic foci among the cells lining the alveoli. These foci were generally closely related to blood capillaries rather than to lymphatics (Fig. 9). Lesions of this order of size (less than 1 mm. in diameter) are not readily visible at autopsy. Their point of origin can be determined by examining serial sections when the majority are found to be separated from the pleura by normal lung. In contrast those early lesions which arise in alveoli in contact with the pleura are generally associated with points at which the pleural network of lymphatics communicates with the interalveolar lymphatics (Fig. 8*a* and *b*).

In suitably stained sections the elastica of interalveolar septa was found to be intact in early lesions. In more advanced lesions we frequently saw areas of hyperplasia and adenoma in the same lung, either as distinct or mixed lesions. In such cases the deficiency of elastica in the adenomatous areas and its almost

---

### EXPLANATION OF PLATES

FIG. 1.—Male BALB/c mouse, 1150/24, age 469 days. H. & E. × 35. Fifty bi-weekly subcutaneous injections = 100 mg. isoniazid (INH). Papillary adenoma originating in subpleural alveoli. Note wedge-shape of neoplastic area corresponding with anatomical sector of lung bounded by pleural lymph follicle and communicating interalveolar lymphatics.

FIG. 2.—Female BALB/c mouse, 1201/7, age 495 days. H. & E. ×6. Single subcutaneous injection of 0·2 mg. 4-nitroquinoline-N-oxide (NQNO), in 0·2 ml. tri-*n*-butyrin. Tumours in subpleural and other sites. Section cut in plane of points of contact with ribs. Note symmetrical distribution of tumours corresponding with pleural lymph follicles.

FIG. 3.—Male $C3H_f$ mouse, 195/59, age 700 days. ×3·5. Untreated control. Note spontaneous tumours and subpleural soot deposits. One tumour in R. lower lobe has a black halo due to invasion of pigment-laden pleural lymph follicle.

FIG. 4.—Male $C3H_f$ mouse, 195/59, as above. H. & E. ×45. Section through tumour with black halo.

Note : (*a*) Pleural lymph follicle with soot-laden macrophages.
(*b*) Subpleural papillary adenoma.
(*c*) Interalveolar lymphatics with soot-laden macrophages.

FIG. 5 (*a*).—Male BALB/c mouse, 1163/48, age 467 days. Van Giesen and elastin. ×8. INH injected as in 1150/24 (Fig. 1). Note invasion of two bronchi and absence of lymphatic engorgement.

(*b*) Intrabronchial extension of pulmonary adenoma. Note black lines of elastin in blood vessels and extending into primary tumour, extreme low centre. ×40.

(*c*) Part of (*b*). Note well differentiated adenoma but no trace of elastin in stroma. ×390.

FIG. 6.—Male BALB/c mouse, 1150/26, age 469 days. H. & E. ×42. INH injected as for 1150/24 (Fig. 1). Note normal lung compressed between tumour and pleura, top right ; and absence of lymphatic engorgement.

FIG. 7*a*.—Male BALB/c mouse 68, age 476 days. ×4. Untreated breeder. Lungs compressed in plastic petri dish. Photographed by surface illumination. Note large black spot (*a*) in L. lobe (upper left) and pale tumour (*b*) with faint grey halo in R. upper lobe (right).

(*b*) Same specimen photographed by transillumination. Note black spot (*a*) now shows presence of translucent tumour.

FIG. 8.—Female BALB/c mouse, 1164/51, age 496 days. Van Giesen and elastin. ×50. (*a*) Injected as for 1150/24 (Fig. 1). (*b*) Marked section of Fig. 8*a*. ×520. Note subpleural origin of tumour and association with lymphatics. Elastic fibres are dense in pleural lymph follicle and interalveolar septa. Communicating lymphatics packed with lymphocytes, shown by arrows.

FIG. 9.—Male BALB/c mouse. H. & E. ×35. Single intraperitoneal injection of 1,2 : 5,6-dibenzanthracene (Table I). Note perivascular arrangement of alveolar hyperplasia and absence of lymphatic involvement.

FIG. 10.—Male $C3H_f$ mouse, 1240/53, age 732 days. INH in drinking water for 674 days ; total dose 2 g. Van Giesen and elastin stain. ×350. Photographed through blue filter to emphasise elastin and reduce nuclear density. Note intact elastic structure through area of alveolar hyperplasia.

FIG. 11.—Same mouse as in Fig. 10. Picro-Mallory and elastin stain. ×350. Note normal distribution of elastin (thin black lines) in areas of alveolar hyperplasia (top and bottom) and absence in neoplastic area (centre). Coarse black lines are due to collagen.

FIG. 12.—Female BALB/c mouse, age 84 days. H. & E. ×42. Single intraperitoneal injection of 1,2 : 7,8-dibenzanthracene (Table I). Subpleural papillary adenoma invading pleural lymph follicles. Note lipping of follicle at both borders of tumour (arrows) and engorgement of interalveolar lymphatics on both sides of tumour. The rest of the lung shows no lymphatic involvement.

1

3

2

4

Peacock and Peacock.

5a

6

5b

7a

5c

7b

8a

8b

9

10

11

12

complete absence in carcinomatous areas, was very striking (Fig. 11). For example, when an adenoma extends into a bronchus, as seen in Fig. 5*a*, *b* and *c*, there is no elastic tissue in the intrabronchial tumour although it is quite well differentiated. Similarly in four clones of transplanted tumours derived from a single spontaneous pulmonary alveologenic carcinoma (Peacock, 1965*b*) the only elastic tissue in the transplants is that of the nutrient blood vessels. Deficiency of elastica in pulmonary tumours in CBA mice was noted by Selbie and Thackray (1948).

We think that this is a point of importance in distinguishing between hyperplastic and neoplastic lesions and gives some indication of the degree of malignancy in the case of progressive neoplasia. The lesions that we classify as hyperplastic are those in which a complete elastic structure similar to that present in the normal alveolar septa is present throughout (Fig. 10). When parts of the lesion show defective or absent elastic tissue, the tumour is classified as an adenoma or carcinoma (Fig. 11). Thus if serial sections show only hyperplasia as defined above, the lesion is so graded. If part of the lesion is neoplastic, it is graded as adenoma or carcinoma. If separate neoplastic and hyperplastic foci are present, each is recorded under the appropriate heading. The total number of lesions in a single lung is not recorded since many are confluent and their enumeration becomes unrealistic, as pointed out by Stewart (1953).

In the case of the young mice in Group 1, injected with carcinogenic hydrocarbons, all but one of the lesions was of the centrally situated hyperplastic variety (Fig. 9 and Table I). The one exception was a subpleural papillary adenoma (Fig. 12 and Table I). The mice injected with non-carcinogenic hydrocarbons and the untreated controls killed at 12 weeks of age showed no hyperplastic or neoplastic pulmonary lesions.

The older mice, Group 2, showed the same range of histological changes but more adenomatous and carcinomatous tumours occur, as might be expected (Table II). However, even in two-year-old mice, sites of early hyperplasia of the alveolar epithelium similar to that seen in the youngest mice, were also observed.

TABLE II.—*Incidence of Alveolar Hyperplasia or Neoplasia in BALB/c Mice Surviving up to 2 Years Receiving Isoniazid Daily Compared with Untreated Controls*

| | Hyperplasia | | Neoplasia | |
|---|---|---|---|---|
| Treatment | Subpleural | Other sites | Subpleural | Other sites |
| Daily INH, 1–2 mg. per mouse | 4/26 | 7/26 | 11/26 | 13/26 |
| Untreated | 11/45 | 4/45 | 16/45 | 14/45 |

The terms " subpleural " and " other sites " refer to the points of origin of lesions, most of which appear at autopsy as subpleural tumours.
Results shown as mice with lesions/mice at risk.

### DISCUSSION AND CONCLUSIONS

Most authors are agreed that the majority of pulmonary tumours found at autopsy are subpleurally situated. This has also been our experience, yet as we have shown, the majority of them do not originate in subpleural sites (Tables I and II). In the case of experimentally administered carcinogens given orally or by parenteral injection, the route of approach to the lung must be through the pulmonary artery (*vis a tergo*). In the case of airborne carcinogens the approach would be to the exposed surface of the alveolar epithelium (*vis a fronte*). We have no clear indication in the case of spontaneous tumours as to which, or either, of these routes may have been involved. However, the presence of soot, either free or more often after phagocytosis in the peripheral alveoli and lymphatics, suggests that airborne carcinogens might be expected to occur in similar situations. In the old mice in Group 2, the distribution of hyperplastic and neoplastic lesions of subpleural origin and of other sites is similar in experimental and control animals.

The results are shown in Table II, indicating only the number of mice affected by the particular type of lesion at subpleural or other sites, but it may be mentioned that lesions of other sites greatly out-number those of subpleural origin.

Other aspects of the INH results will be more fully discussed elsewhere. For present purposes, only their histogenesis and site of origin are considered. It seems probable that tumours of subpleural origin in both experimental and control groups are partly accounted for by the same aetiological factors, which probably include airborne carcinogens.

It is concluded that alveolar hyperplasia in the mouse lung is an early manifestation of an essentially neoplastic process. The degree of neoplasia can be objectively determined by examining serial sections stained for elastin. In hyperplasia with normal distribution of elastin the lesion may be regarded as possibly transient but when elastin is progressively lost as neoplasia proceeds, it seems unlikely that the lesion could retrogress.

It is considered important that the site of origin of all types of hyperplasia and neoplasia of alveolar epithelium should be recorded in relation to the factors discussed above. It seems reasonable that blood-borne factors (*vis a tergo*) would tend to cause lesions at the point of maximum impact in relation to the terminal capillaries of the pulmonary artery, whereas airborne factors (*vis a fronte*) might be expected to affect the alveolar epithelium of any part of the lung ; but the evidence for the association particularly of tumours of subpleural origin with engorgement of peripheral lymphatics suggests that the concentration of airborne carcinogens at these sites may be of aetiological importance.

### SUMMARY

Induced and spontaneous pulmonary alveolar hyperplasia and neoplasia in young and old mice are compared.

Most pulmonary tumours large enough to be visible to the naked eye appear to be subpleural but distinction should be drawn between those of subpleural and those of deeper origin.

A classification based on anatomical site of origin and histological criteria is proposed.

Subpleural hyperplasia and neoplasia are associated with the sites of normal subpleural lymph follicles and the communicating interstitial lymphatics of the lung which often show local engorgement with lymphocytes.

Histologically similar lesions without lymphatic involvement occurring at sites other than subpleural are associated with terminal pulmonary arterioles.

Hyperplastic and neoplastic lesions can be distinguished by the amount and arrangement of supporting elastica which is normal in hyperplastic and defective or absent in neoplastic lesions.

It is suggested that the subpleural lesions are caused mainly by airborne carcinogens (*vis a fronte*) and the lesions at other sites by blood-borne carcinogens (*vis a tergo*).

One of us (P. M. P.) was in receipt of a full-time grant from the British Empire Cancer Campaign for Research during the early part of this work.

### REFERENCES

HESTON, W. H.—(1940) *J. natn. Cancer Inst.*, **1**, 109.
LIVINGOOD, L. E.—(1896) *Johns Hopkins Hosp. Bull.*, **7**, 177.
PEACOCK, P. R.—(1965*a*) Quadrennial International Conference on Cancer, Perugia, **3**, (in press).
PEACOCK, P. R.—(1965*b*) *J. Path. Bact.*, **89**, 285.
PIETRA, G., SPENCER, K. AND SHUBIK, P.—(1959) *Nature, Lond.*, **183**, 1689.
ROE, F. J. C., ROWSON, K. E. K. AND SALAMAN, M. H.—(1961) *Br. J. Cancer*, **15**, 515.
SELBIE, F. R. AND THACKRAY, A. C.—(1948) *Br. J. Cancer*, **2**, 380.
STEWART, H. L.—(1953) In ' Physiopathology of Cancer '. London (Cassell), p. 93.
STRONG, L. C.—(1936) *J. Hered.*, **27**, 21.


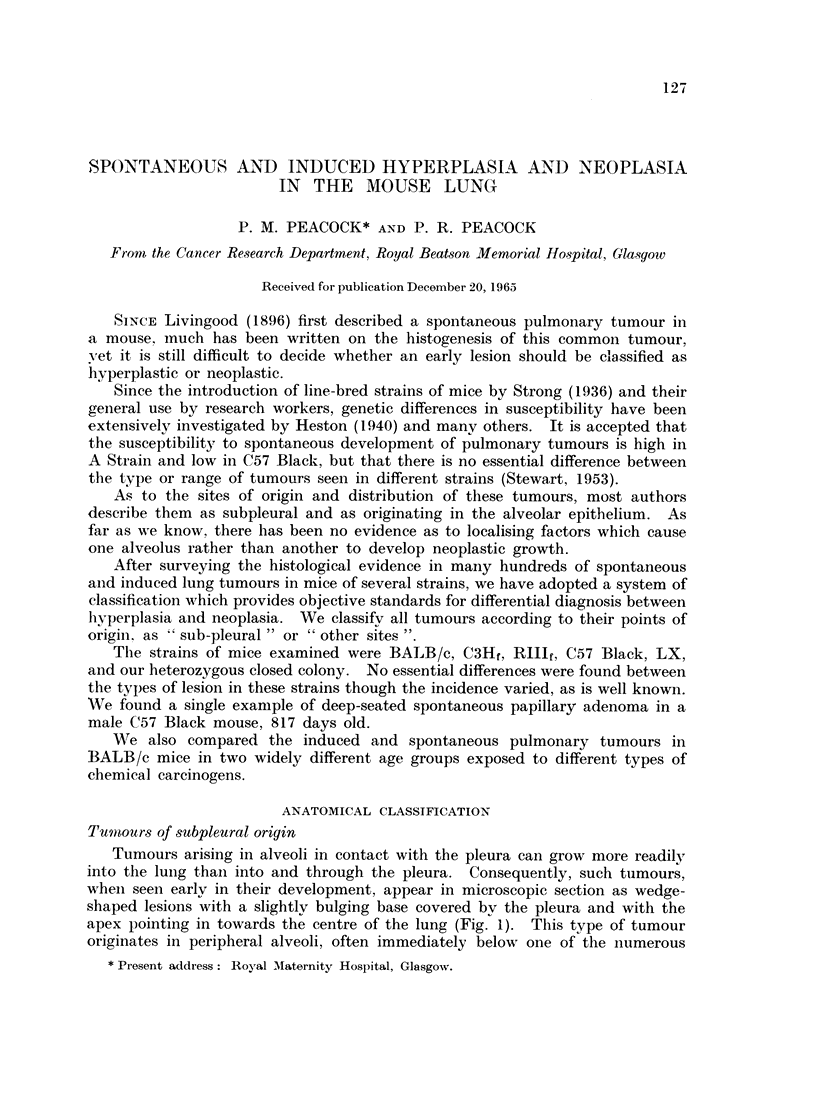

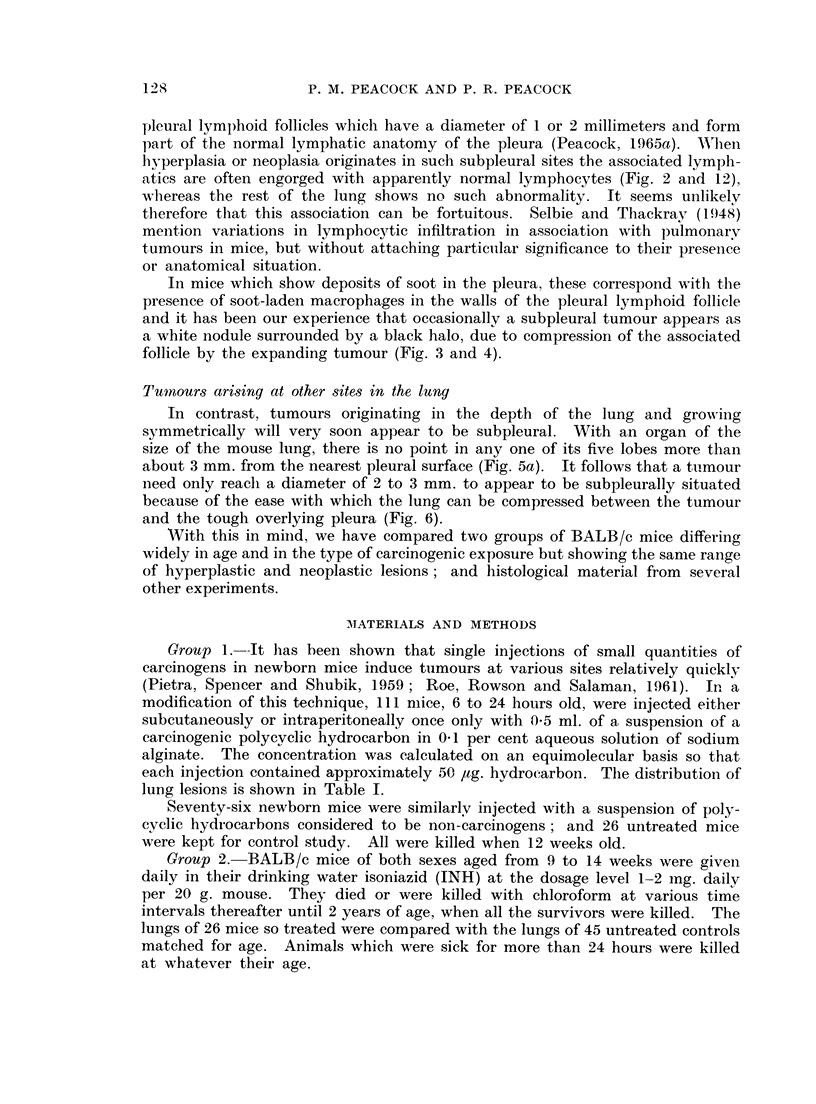

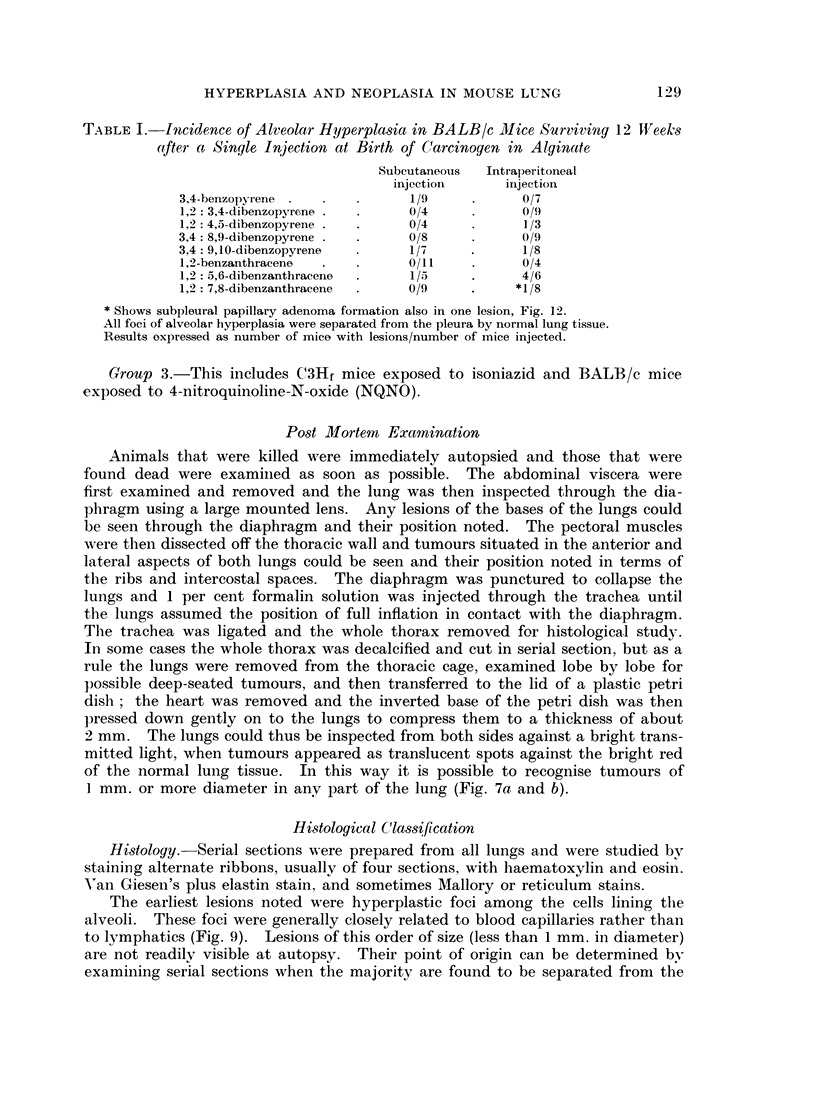

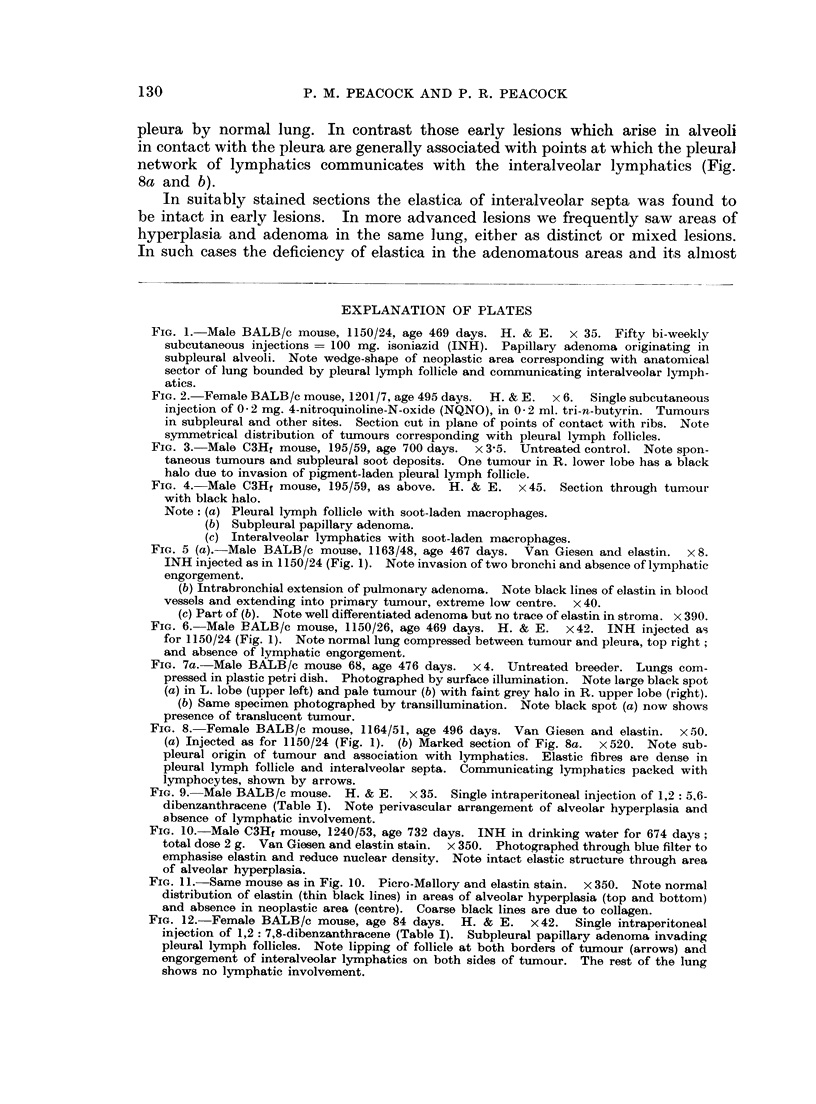

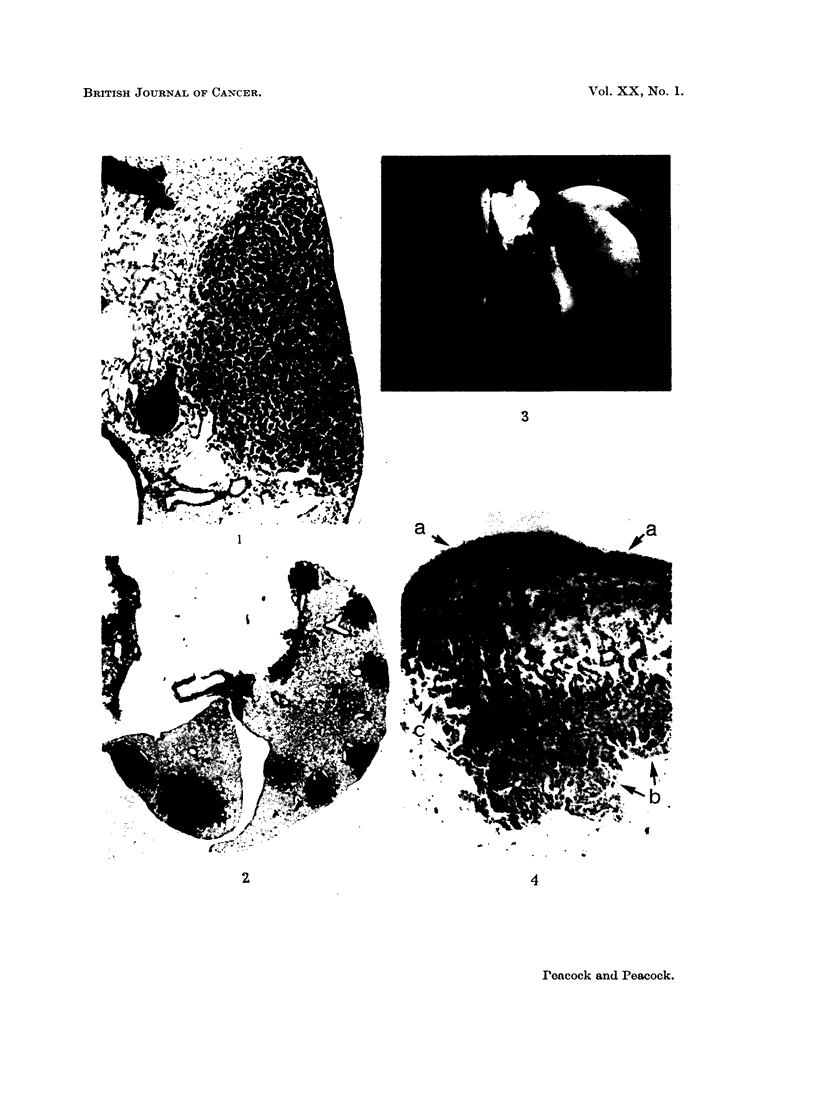

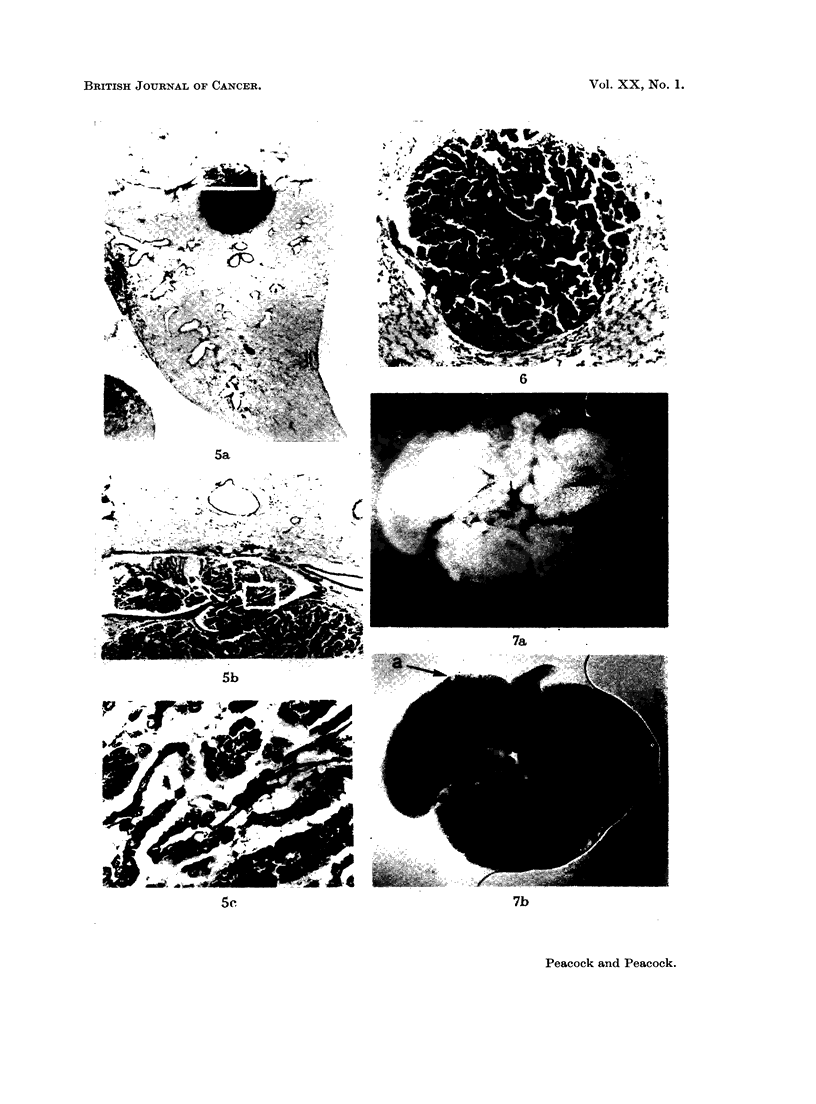

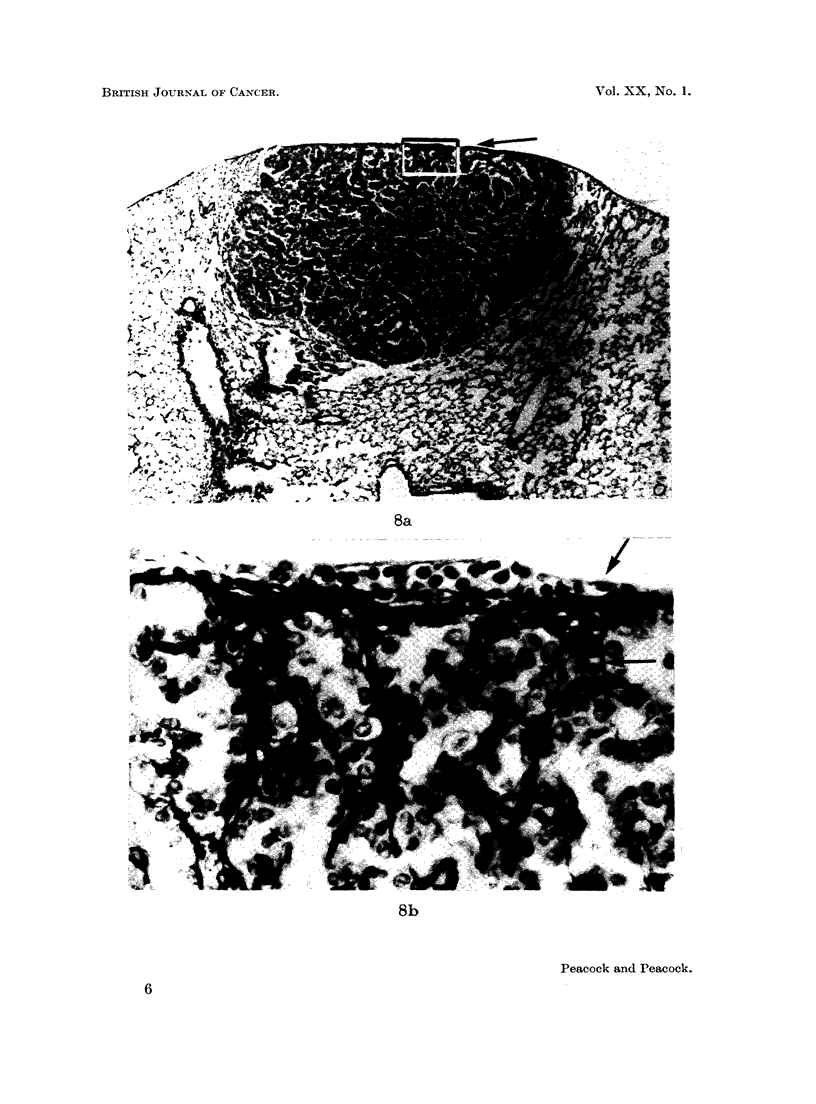

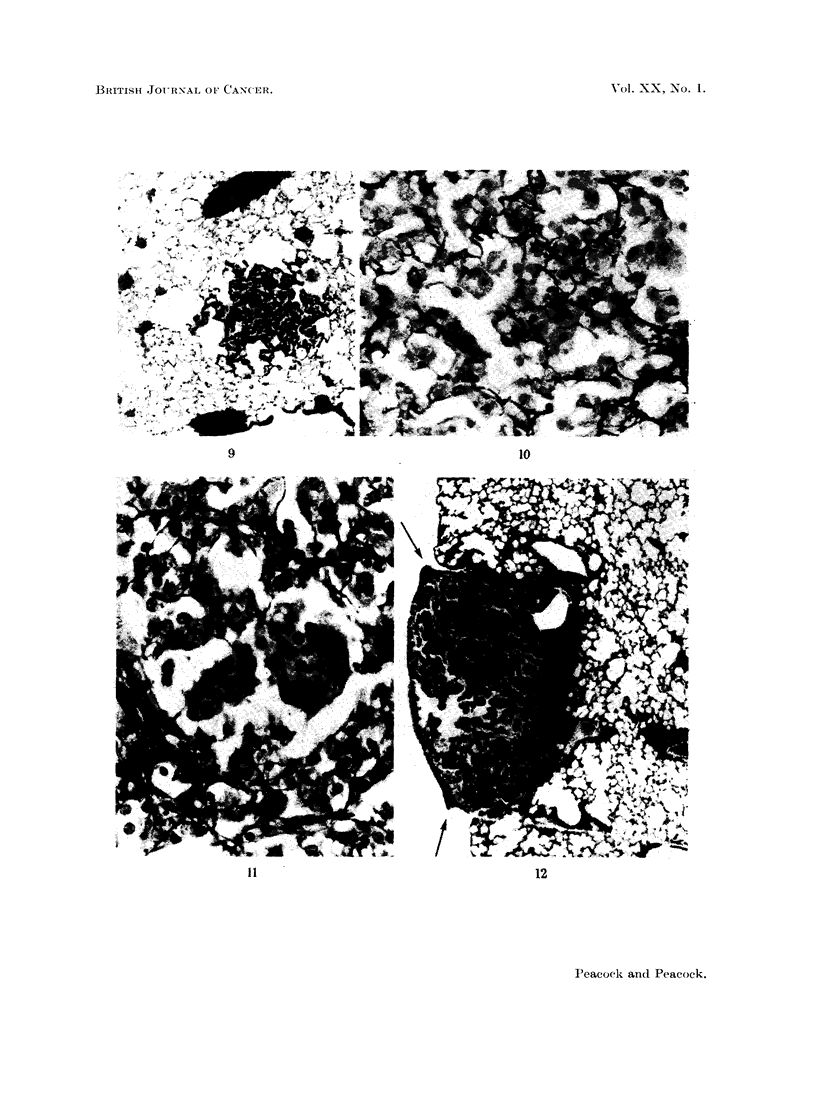

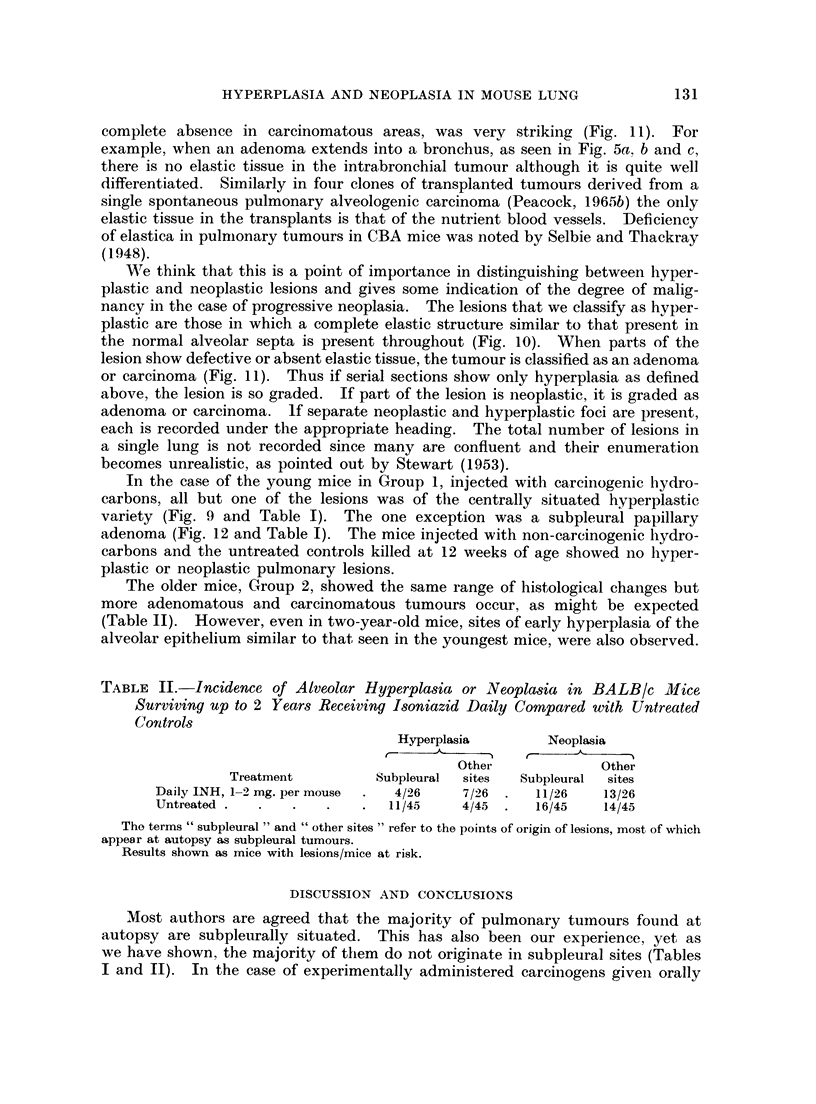

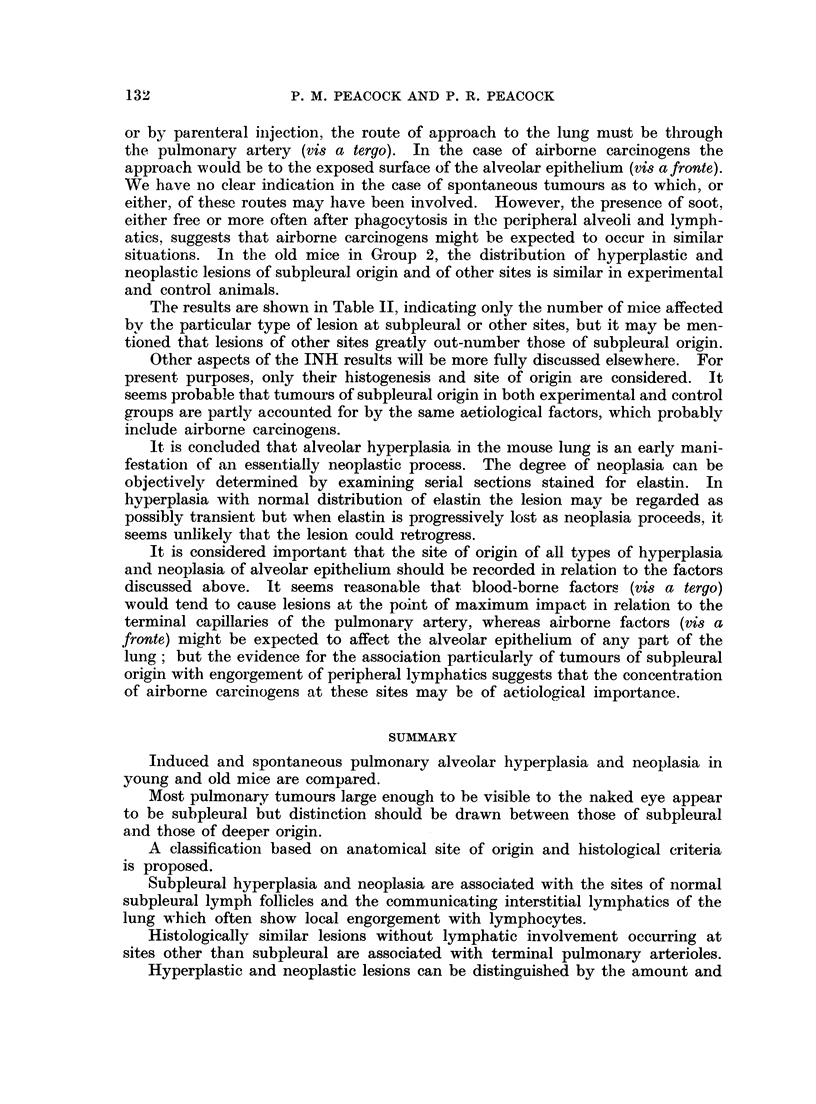

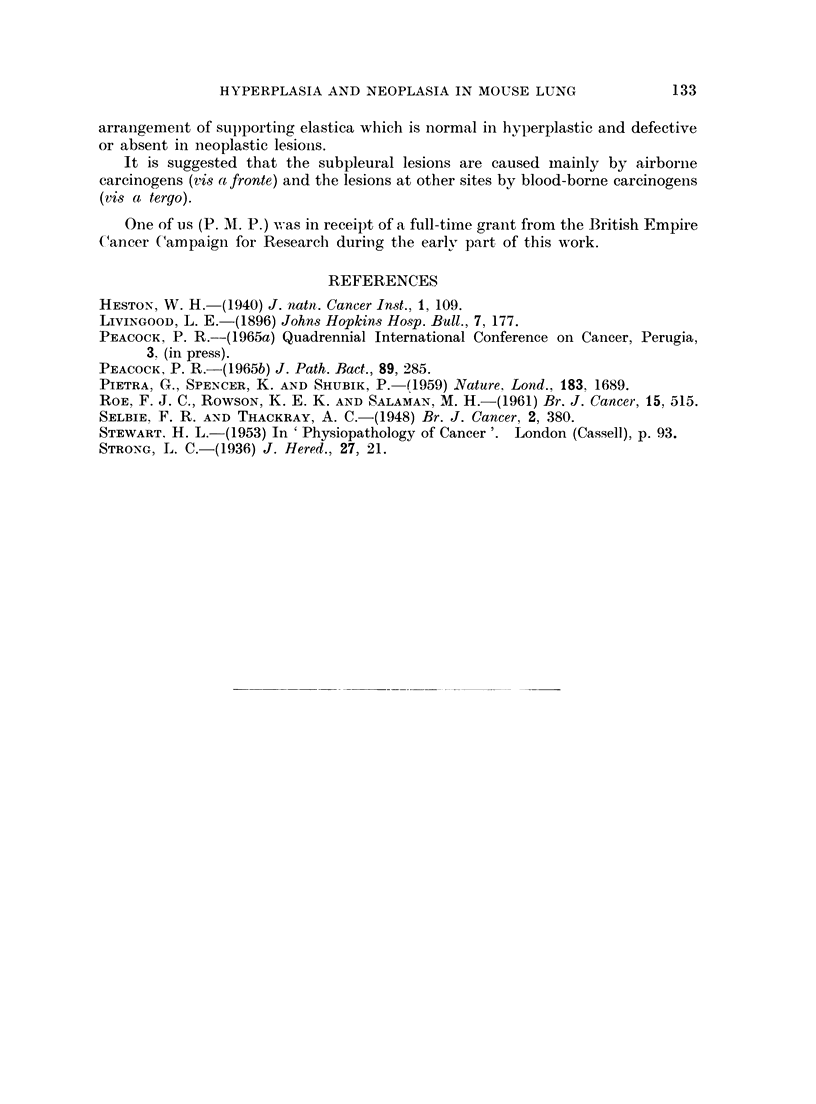


## References

[OCR_00563] PIETRA G., SPENCER K., SHUBIK P. (1959). Response of newly born mice to a chemical carcinogen.. Nature.

[OCR_00565] ROE F. J., ROWSON K. E., SALAMAN M. H. (1961). Tumours of many sites induced by injection of chemical carcinogens into newborn mice, a sensitive test for carcinogenesis: the implications for certain immunological theories.. Br J Cancer.

